# Electrochemotherapy Following Surgical Cytoreduction for the Treatment of Recurrent Equine Sarcoids: A Retrospective Study of 23 Lesions

**DOI:** 10.3390/vetsci13070691

**Published:** 2026-07-16

**Authors:** Alessandro Spadari, Federica Meistro, Raimondo Tornago, Cristian Facchetti, Paola D’Angelo, Maria Virginia Ralletti, Salvatore Montemagno, Riccardo Rinnovati

**Affiliations:** 1 Department of Veterinary Medical Sciences, University of Bologna, Ozzano dell’Emilia, 40064 Bologna, Italy; alessandro.spadari@unibo.it (A.S.); federica.meistro@unibo.it (F.M.); paola.dangelo7@unibo.it (P.D.); virginia.ralletti@unibo.it (M.V.R.); salvatore.montemagn2@unibo.it (S.M.); riccardo.rinnovati2@unibo.it (R.R.); 2 Independent Researcher, 39012 Merano, Italy; raimondotornago@gmail.com

**Keywords:** equine sarcoid, electrochemotherapy, electroporation, horse, recurrent sarcoid, veterinary oncology

## Abstract

Despite the wide range of therapeutic options currently available for equine sarcoids, recurrence of these lesions remains a major clinical challenge. Consequently, the identification of effective strategies for the management of recurrent sarcoids remains an important objective in equine oncology. The aim of this retrospective study was to evaluate the clinical outcome of surgical cytoreduction combined with intraoperative electrochemotherapy in horses affected by recurrent equine sarcoids. Medical records of horses treated between July 2018 and August 2025 at the Veterinary Teaching Hospital of the University of Bologna were retrospectively reviewed. Data collected included signalment, lesion location, clinical subtype, lesion size, chemotherapeutic agent used, recurrence status, and follow-up duration. Fourteen horses presenting 23 recurrent sarcoid lesions met the inclusion criteria. Recurrence was evaluated at both the lesion and horse levels. Local tumour control was achieved in 16 of 23 lesions (69.6%). Recurrence was observed in 7 lesions, corresponding to a lesion-based recurrence rate of 30.4%, and in 5 of 14 horses (35.7%). Malignant and fibroblastic lesions showed higher recurrence rates than other clinical subtypes, whereas larger lesions tended to recur more frequently than smaller lesions. No significant differences were identified between cisplatin- and carboplatin-treated lesions. Surgical cytoreduction combined with intraoperative electrochemotherapy provided encouraging local tumour control in a population composed exclusively of recurrent sarcoids. This treatment protocol may represent a valuable therapeutic option for horses with local recurrence of sarcoids, although larger prospective studies are required to further define prognostic factors and long-term outcomes.

## Simple Summary

Equine sarcoids are the most common skin tumours in horses and are well known for their tendency to recur after treatment. Recurrent sarcoids represent a particularly challenging clinical problem because they have persisted or recurred despite one or more previous therapeutic interventions and often become increasingly difficult to manage. Electrochemotherapy combines local administration of chemotherapics agents with short electrical pulses that enhance drug uptake by tumour cells. The aim of this study was to evaluate the outcome of surgical cytoreduction followed by intraoperative electrochemotherapy in horses affected by recurrent sarcoids. Fourteen horses with 23 recurrent lesions were included. Local tumour control was achieved in the majority of treated lesions, with recurrence observed in 30.4% of cases during follow-up. Larger lesions and more aggressive clinical forms tended to recur more frequently. These findings suggest that surgical cytoreduction combined with electrochemotherapy may represent a useful treatment option for recurrent equine sarcoids, particularly in cases where previous therapies have failed.

## 1. Introduction

Equine sarcoids (ES) are the most common skin neoplasms in horses, and account for a substantial proportion of all equine cutaneous tumours worldwide [1]. Although these fibroblastic tumours do not metastasize, they are characterized by locally invasive behaviour, frequent recurrence following treatment, and considerable therapeutic challenges [2]. Depending on their size, anatomical location, form and clinical presentation, ES may negatively affect horse welfare and athletic performance, often resulting in prolonged treatment courses and significant economic consequences for owners [3].

The aetiopathogenesis of ES has been strongly associated with infection by bovine papillomavirus (BPV), particularly BPV-1 and BPV-2. Viral DNA has been consistently identified within sarcoid tissues, supporting a central role of papillomavirus infection in tumour development. However, BPV infection alone appears insufficient to induce tumour formation, and additional factors, including host genetic susceptibility, local trauma, immune dysfunction, wound-healing abnormalities, and environmental influences, are believed to contribute to disease development and progression [4,5,6,7].

Clinically, sarcoids are classified into six forms based on their gross appearance: occult, verrucous, nodular, fibroblastic, mixed, and malignant [2,8]. These categories do not necessarily represent distinct biological entities, and lesions may evolve from one form to another over time, particularly following trauma or unsuccessful treatment attempts. Moreover, the considerable clinical heterogeneity and the frequent overlap between different morphological forms may complicate accurate lesion classification, potentially influencing treatment selection and contributing to variable therapeutic outcomes. This biological variability, combined with the unpredictable behaviour of individual lesions, contributes substantially to the difficulty of achieving long-term tumour control [8].

A wide variety of treatment options have been described, including surgical excision, laser surgery, cryotherapy, radiotherapy, immunotherapy, molecular approach, intralesional chemotherapy, topical cytotoxic agents, and combination protocols [9,10,11]. However, no single treatment has consistently demonstrated universal effectiveness. Reported recurrence rates remain highly variable across studies and are influenced by factors such as lesion size, anatomical location, clinical subtype, treatment modality, and previous treatment history [12,13].

Among these factors, previous therapeutic failure is widely recognized as one of the strongest predictors of subsequent recurrence. Recurrent equine sarcoids (RES) frequently represent a more challenging clinical condition than primary lesions because repeated surgical manipulation, chronic inflammation, scar formation, and residual microscopic disease may alter local tissue architecture and complicate further treatment attempts [12,13,14].

The management of RES therefore remains one of the most challenging areas in equine oncology. A major limitation of the current literature is that outcomes for primary and recurrent lesions are frequently analysed together, making it difficult to accurately assess treatment efficacy in horses presenting with treatment-resistant disease. Furthermore, comparisons among available therapies are complicated by marked heterogeneity in study populations, lesion characteristics, and treatment protocols. Consequently, there is currently insufficient evidence to determine whether specific therapeutic approaches are more effective for particular clinical forms of sarcoids or for recurrent rather than primary lesions [15].

For these reasons, there is a growing interest in therapeutic strategies capable of improving local tumour control while minimizing damage to surrounding tissues, particularly in recurrent lesions for which previous interventions have failed. Electrochemotherapy (ECT) is a local antitumour treatment that combines the administration of chemotherapeutic agents with the application of short, high-intensity electric pulses. These pulses induce reversible electroporation of tumour cell membranes, resulting in a marked increase in intracellular drug uptake and enhanced cytotoxic efficacy. In addition to its direct antitumour effect, ECT induces transient vascular disruption and a vascular-lock phenomenon, which further contribute to tumour control by reducing blood flow within treated tissues [16]. Over the last decade, chemotherapy has gained increasing attention in both human and veterinary oncology and has shown promising results in the treatment of ES [17,18,19]. The combination of surgical cytoreduction followed by intraoperative ECT has been proposed as a particularly attractive strategy for locally invasive or recurrent lesions. Surgical debulking reduces tumour burden, while ECT may improve eradication of residual neoplastic cells that remain beyond the visible surgical margins, potentially reducing the risk of local recurrence [15,20].

Despite these encouraging reports, relatively limited information is available regarding the effectiveness of ECT specifically in RES. Furthermore, factors potentially associated with treatment failure in this subgroup remain poorly defined.

Therefore, the primary aim of this retrospective study was to evaluate the clinical outcome and local tumour control achieved following surgical cytoreduction combined with intraoperative ECT in horses affected by RES.

Secondary objectives were to determine the recurrence rate following treatment, to explore differences in clinical outcomes between cisplatin- and carboplatin-based ECT protocols, and to investigate potential associations between tumour recurrence and selected clinical variables, including lesion size, anatomical location, sarcoid classification, and previous treatment history. In addition, we sought to explore whether specific sarcoid subtypes exhibited distinct patterns of biological behaviour or treatment response, and to identify clinical characteristics potentially associated with increased treatment resistance.

We hypothesized that the combination of surgical cytoreduction and ECT would provide satisfactory local tumour control despite the challenging nature of RES, and that larger lesions, particular subtypes, extensive previous treatment history, and specific anatomical locations would be associated with an increased risk of recurrence.

## 2. Materials and Methods

### 2.1. Study Design and Case Selection

This retrospective study was conducted at the Veterinary Teaching Hospital of the Department of Veterinary Medical Sciences, University of Bologna, Italy. Medical records of horses presented for treatment of RES between July 2018 and August 2025 were reviewed. Follow-up information was subsequently collected from hospital records, clinical re-examinations, referring veterinarians, and owner interviews up to June 2026.

Cases were identified by searching the hospital medical record database using the terms “sarcoid”, “electrochemotherapy”, “electroporation”, “cisplatin”, and “carboplatin”. Retrieved records were manually reviewed to verify eligibility and completeness of clinical information.

For the purposes of this study, RES were defined as persistent or recurrent tumour growth arising within or immediately adjacent to the site of a previously treated sarcoid after one or more therapeutic interventions. The development of a new sarcoid at a distant anatomical location was not considered recurrence. Previous treatments included surgical excision, cryotherapy, laser surgery, intralesional chemotherapy, topical therapies, immunomodulatory treatments, or combinations thereof.

To be eligible for inclusion, horses were required to meet all of the following criteria: (1) a clinical diagnosis of ES established by experienced equine surgeons on the basis of lesion appearance, anatomical distribution, histological diagnosis and clinical history; (2) recurrent disease according to the study definition; (3) treatment performed at the University of Bologna Veterinary Teaching Hospital using the combined protocol of surgical cytoreduction and ECT; and (4) availability of adequate clinical documentation, including signalment, lesion characteristics, treatment records, and post-treatment follow-up information.

Cases were excluded when the diagnosis of sarcoid was uncertain, when treatment protocols differed from the combined surgical cytoreduction and ECT approach evaluated in this study, when essential clinical information was unavailable, or when no follow-up data could be obtained. Lesions receiving additional adjunctive therapies during the study period that could substantially influence outcome assessment were also excluded from statistical evaluation.

### 2.2. Data Collection

For all horses meeting the inclusion criteria, data were extracted from the medical records and entered into a standardized electronic database developed in Microsoft Excel (Microsoft Corporation, Redmond, WA, USA). The database was specifically designed to collect horse-level and lesion-level variables considered relevant to treatment outcome and recurrence.

At the horse level, the following information was recorded: breed, sex, age at the time of treatment, date of presentation, number of sarcoid lesions treated during the study period, and previous therapeutic history. Particular attention was given to documenting prior treatment attempts, as all horses included in the study presented with recurrent disease. When available, the type and number of previous treatments were recorded.

Data were also collected at the lesion level. Both single and multiple lesions were included. Each sarcoid was considered an independent observational unit for descriptive and statistical analyses. For each lesion, anatomical location, histologic diagnosis, clinical appearance, dimensions, chemotherapeutic agent used, recurrence status, and follow-up duration were recorded.

Information regarding previous treatment history was collected whenever available from the medical records and owner reports. However, because many horses had been treated at external institutions before referral, detailed information regarding the specific treatment modality employed was not consistently available for all cases. Consequently, previous treatment history was recorded descriptively when documented but was not included in formal statistical analyses.

Anatomical location was categorized according to body region. Lesions were grouped as involving the head, periocular region, ear, cervical region, thorax, abdomen, axillary region, inguinal or genital region, thoracic limbs, pelvic limbs, or perineal region.

Clinical type appearance was classified according to the classification proposed by Knottenbelt [2], distinguishing occult, verrucous, nodular, fibroblastic, mixed, and malignant sarcoids. Classification was based on histologic descriptions and photographic documentation available in the medical records.

Tumour dimensions were obtained from clinical examination records. For statistical analyses, the maximum tumour diameter was used as the principal indicator of lesion size because this variable was consistently available for all lesions and allowed standardized comparison among cases.

The chemotherapeutic agent used during ECT (cisplatin or carboplatin) was allocated randomly before treatment. Allocation was performed independently of horse signalment, lesion size, anatomical location, clinical subtype, or previous treatment history, with the aim of obtaining two comparable treatment groups.

Follow-up information was collected from hospital re-examinations, photographic records, referring veterinarians, and owner interviews. For each lesion, recurrence status was documented as either present or absent. When recurrence occurred, the interval between treatment and detection of tumour regrowth was recorded whenever available.

No surgery-only control group was included, as the aim of the study was to evaluate the clinical outcome of the combined treatment protocol rather than to compare different treatment modalities.

### 2.3. Treatment Protocol

All lesions included in the study were treated using a combined therapeutic approach consisting of surgical cytoreduction immediately followed by intraoperative ECT. All procedures were performed by experienced equine surgeons at the Veterinary Teaching Hospital of the University of Bologna. Horses were all treated under general anaesthesia. Prior to anaesthetic induction, all horses underwent a complete physical examination and routine preoperative assessment to confirm suitability for general anaesthesia. Anaesthetic management followed standard hospital protocols and in accordance with individual patient requirements. Depending on tumour location and surgical accessibility, horses were placed either in dorsal recumbency or lateral recumbency.

Following routine aseptic preparation of the surgical field, the sarcoid lesion was surgically excised using a scalpel blade. The surgical approach was tailored to the size, anatomical location, and extent of each lesion. Particular attention was paid to preserving surrounding anatomical structures while achieving maximal macroscopic tumour removal.

Given the recurrent nature of all lesions included in the study, complete excision with wide oncological margins was frequently limited by anatomical constraints, previous surgical interventions, local fibrosis, and the infiltrative growth pattern typically observed in RES. Therefore, the primary surgical objective was maximal gross tumour debulking rather than radical excision.

Excision was performed by sharp dissection along the interface between the tumour and surrounding tissues whenever identifiable.

After tumour removal, the surgical site was carefully inspected for residual gross disease and haemostasis was achieved using standard surgical techniques.

**Figure 1:**
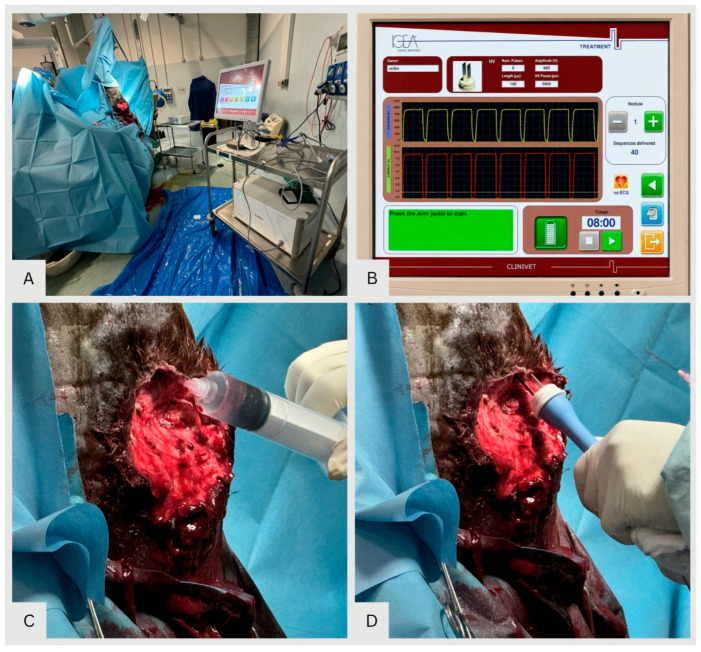
Intraoperative electrochemotherapy procedure following surgical cytoreduction of a recurrent equine sarcoid. (**A**) Intraoperative view of the surgical setting during ECT under general anaesthesia. (**B**) Electroporation device interface displaying treatment parameters used for pulse delivery. (**C**) Local infiltration of the surgical bed with the chemotherapeutic agent following tumour cytoreduction. Multiple injections were performed to ensure homogeneous distribution throughout the treatment field and surrounding tissues at risk for residual microscopic disease. (**D**) Application of electric pulses using needle electrodes immediately after chemotherapeutic infiltration, promoting increased intracellular drug uptake through reversible electroporation and enhancing local tumour control.

ECT was performed immediately after cytoreduction using either cisplatin or carboplatin as the chemotherapeutic agent. Drug selection was random. The chemotherapeutic solution was administered locally within the surgical site and surrounding tissues considered at risk for residual microscopic tumour infiltration (Figure 1C). The selected chemotherapeutic agent (cisplatin or carboplatin) was administered by multiple injections throughout the surgical bed and the surrounding tissues considered at risk for residual microscopic tumour infiltration. The injections of chemotherapeutic agents are spaced and concentrated with the intention of obtaining saturation of the tissues infiltrated. Following drug administration, electric pulses were delivered using an electroporation device equipped with needle electrodes specifically designed for ECT applications (Figure 1D). The electro-porator used for case 1 to 6 was an ELECTRO vet S13 (Leroy Biotech, Saint-Orens-de-Gameville, France) equipped with L-shaped or Needle electrodes. For the other cases the electro-porator used was a CLINIVET (IGEA Medical, Carpi, Italia) equipped with Plate electrodes or Finger electrodes. Both the machines deliver a sequence of 8 pulses lasting 100µs each, with voltage and frequency dependent on the specific presetting of the electrode used. Pulse delivery was performed in accordance with established ECT principles to ensure homogeneous treatment of the entire surgical field. The electrodes were sequentially repositioned as necessary to cover the complete surgical bed together with a margin of adjacent tissue potentially containing residual neoplastic cells.

Following completion of ECT, surgical wound management was determined by the operating surgeon according to the anatomical location, size of the surgical defect, and local tissue tension. In most cases, wounds were left open and allowed to heal by second intention because extensive tissue loss following cytoreduction and local administration of chemotherapeutic agents combined with electroporation could adversely affect early wound healing and increase the risk of wound dehiscence. However, when considered appropriate, particularly in cosmetically or functionally important regions such as the periocular area, partial primary closure was achieved using a limited number of appositional sutures to reduce the wound defect, improve tissue apposition, and preserve normal anatomical conformation.

### 2.4. Postoperative Management

Following recovery from general anaesthesia, horses received routine postoperative care and monitoring during hospitalization. Anti-inflammatory therapy consisted of flunixin meglumine (1.1 mg/kg IV or PO, once daily) administered for 4 days, although minor adjustments were made according to individual clinical requirements and clinician judgement. Systemic antimicrobial therapy was not routinely administered, as the surgical sites were considered clean-contaminated and postoperative wound management relied primarily on local monitoring and second-intention healing.

Because all lesions underwent ECT, surgical wounds were intentionally left open and managed by second-intention healing. No primary wound closure was attempted, and bandages or occlusive dressings were not routinely applied. This approach was adopted to facilitate drainage, allow regular assessment of the treated area, and avoid potential complications associated with closure of tissues exposed to local chemotherapeutic agents.

Postoperative wound care was intentionally conservative. Owners were instructed to perform daily visual inspection of the surgical site and to monitor for excessive swelling, abnormal discharge, exuberant granulation tissue formation, or other signs of local complications. When necessary, gentle cleansing of the wound surface was performed to remove debris and maintain local hygiene.

Particular attention was directed toward insect control during the healing period, especially in lesions located in anatomical regions commonly exposed to flies. Physical protection and topical insect-repellent products were recommended when considered appropriate in order to minimize irritation, contamination, and self-trauma of the healing tissues.

Following discharge, horses were re-evaluated through scheduled clinical examinations, photographic documentation provided by owners, and communication with referring veterinarians when applicable. Monitoring continued until complete wound healing was achieved and subsequently focused on long-term assessment of local tumour control and detection of recurrence.

### 2.5. Follow-Up and Outcome Assessment

Follow-up data were collected retrospectively as stated by the study protocol.

The date of treatment was considered day 0 for all analyses. Follow-up duration was calculated from the date of surgery and ECT to the most recent clinical update available at the time of data collection or to the date on which recurrence was first identified.

The primary outcome variable was local tumour recurrence. Local recurrence was defined as either persistence of the treated lesion without complete clinical resolution or regrowth of a lesion clinically consistent with sarcoid within the original surgical site or in direct continuity with it after treatment. Lesions arising at anatomically distinct sites were considered new primary sarcoid rather than recurrences and were therefore excluded from recurrence analyses. When it occurred, the interval between treatment and recurrence detection was recorded.

Outcome assessment was performed both at the lesion level and at the horse level. For lesion-based analyses, each treated sarcoid was considered an independent observational unit and classified as either recurrent or non-recurrent. For horse-based analyses, a horse was considered recurrent when at least one treated lesion developed recurrence during the follow-up period. This dual approach was adopted because individual horses frequently presented with multiple lesions differing in anatomical location, clinical subtype, size, and previous treatment history, all of which could reasonably influence biological behaviour and treatment response despite receiving the same treatment protocol. Lesion-level analyses were therefore performed to investigate associations between lesion-specific characteristics and outcome, whereas horse-level analyses were included to provide an overall patient-based assessment of treatment success. In addition to recurrence status, the duration of tumour-free follow-up was recorded for all lesions whenever possible. Lesions showing no evidence of recurrence at the last available follow-up examination were classified as locally controlled at the time of final assessment.

To ensure consistency of outcome classification, available clinical records and photographic documentation were independently reviewed by the investigators whenever recurrence status was uncertain. Cases lacking sufficient information to confidently determine outcome were excluded from analyses requiring definitive recurrence classification.

### 2.6. Statistical Analysis

Statistical analyses were performed to provide both a descriptive overview of the study population and an exploratory evaluation of factors potentially associated with tumour recurrence following treatment.

Descriptive statistics were initially used to summarize horse- and lesion-related characteristics. Continuous variables, including age, lesion dimensions, and follow-up duration, were assessed for normality and reported as mean ± standard deviation or median and range, as appropriate. Categorical variables, including sex, breed, anatomical location, clinical appearance, chemotherapeutic agent used, and recurrence status, were summarized as frequencies and percentages.

Treatment outcome was evaluated at both the lesion level and the horse level. Lesion-based recurrence rate was calculated as the proportion of treated sarcoids that developed recurrence during the follow-up period. Horse-based recurrence rate was calculated as the proportion of horses presenting recurrence of at least one treated lesion at the same anatomical location.

Exploratory analyses were performed to investigate possible associations between recurrence and selected clinical variables. Given the limited sample size and the non-parametric nature of several variables, Fisher’s exact test was used to evaluate associations between recurrence and categorical variables, including anatomical location, clinical classification of the sarcoid, and chemotherapeutic agent administered during ECT. Differences in lesion size between recurrent and non-recurrent lesions were evaluated using the Mann–Whitney U test.

Because of the retrospective design of the study and the relatively small number of observations available for some categories, statistical analyses were considered exploratory rather than confirmatory. Consequently, the primary focus of the study remained the descriptive evaluation of clinical outcome and local tumour control. Statistical significance was set at *p* < 0.05.

All statistical analyses were performed using GraphPad Prism version 10.2.3 for macOS (GraphPad Software, Boston, MA, USA). 

### 2.7. Ethical Statement

This study was retrospective and involved analysis of clinical data from horses treated as part of routine veterinary care. No experimental procedures were performed for the purpose of the study. Ethical approval was therefore not necessary according to institutional regulations. Owner consent for treatment was obtained at the time of hospital admission.

## 3. Results

### 3.1. Study Population

A total of 14 horses presenting 23 recurrent equine sarcoid lesions met the inclusion criteria and were included in the study. The population consisted of 8 mares (57.1%) and 6 geldings (42.9%), with a median age of 10.5 years (range 5–21 years) at the time of treatment.

A variety of breeds were represented, including Italian Saddle Horses (n = 3), Purebred Arabians (n = 2), Haflingers (n = 2), donkeys (n = 2), and one horse each of the following breeds: Quarter Horse, Paint Horse, Selle Français, Appaloosa, and KWPN.

Most horses presented with a single lesion (8/14, 57.1%), whereas 3 horses (21.4%) presented with two lesions and 3 horses (21.4%) presented with three lesions. The median number of treated lesions per horse was 1 (range 1–3).

Considering the limited sample size and the exploratory nature of the statistical analyses, the observed associations should be interpreted with caution and regarded as hypothesis-generating rather than confirmatory.

### 3.2. Lesion Characteristics

A total of 23 RES lesions were treated during the study period. Lesions were distributed across multiple anatomical regions.

The most frequently affected anatomical location was the genitomammary region (6/23, 26.1%), followed by the pelvic limbs (5/23, 21.7%), thoracic limbs (3/23, 13.0%), periocular region (2/23, 8.7%), ears (2/23, 8.7%), and perineal region (2/23, 8.7%). Single lesions were recorded in the head, cervical region, and thoracic/abdominal region.

Clinical appearance varied considerably among lesions. Nodular sarcoids represented the most common subtype (9/23, 39.1%), followed by fibroblastic lesions (6/23, 26.1%), verrucous lesions (4/23, 17.4%), malignant lesions (3/23, 13.0%), and occult lesions (1/23, 4.3%).

Lesion size was highly variable. The median maximum lesion diameter was 5.0 cm (range 1.5–25.0 cm), reflecting the inclusion of both relatively small lesions and extensive recurrent masses.

The exact number of previous therapeutic interventions was documented for only eight lesions. Of these, six lesions (26.1%) had received one previous treatment before referral, whereas two lesions (8.7%) had undergone two previous treatment attempts.

With regard to the ECT protocol, cisplatin was used in 12 lesions (52.2%), whereas carboplatin was administered in 11 lesions (47.8%). 

### 3.3. Clinical Outcome and Recurrence Rate

Follow-up information was available for all lesions included in the study. Overall, local tumour control was achieved in the majority of treated sarcoids. Recurrence was documented in 7 of the 23 treated lesions, corresponding to a lesion-based recurrence rate of 30.4%. Conversely, 16 lesions (69.6%) showed no evidence of recurrence during the available follow-up period.

At the horse level, recurrence of at least one treated lesion occurred in 5 of the 14 horses included in the study (35.7%), whereas 9 horses (64.3%) remained free from recurrence at all treated sites throughout follow-up (Table 1).

Follow-up duration varied considerably among cases. Median follow-up was 420 days (range 40–2877 days). Among lesions that remained recurrence-free, the median tumour-free follow-up period was 630.5 days (range 306–2877 days), indicating long-term local control in many treated cases. Among recurrent lesions, the median time to recurrence was 105 days (range 40–431 days). Most recurrences were identified within the first months following treatment, although later recurrences were also observed in a limited number of cases.

All surgical wounds healed by second intention according to the treatment protocol. No major postoperative complications requiring additional surgical intervention were identified in the available medical records.

### 3.4. Recurrence According to Lesion Characteristics

Recurrence was observed across multiple anatomical locations. Pelvic limb lesions showed the highest number of recurrences, accounting for 3 of the 7 recurrent lesions identified during follow-up. Additional recurrences were observed in the thoracic limb, periocular region, genitomammary region, and thoracic/abdominal region. When lesions were grouped according to anatomical distribution, recurrence occurred in 4 of 8 limb-associated lesions (50.0%) and in 3 of 15 lesions located elsewhere (20.0%). Although recurrence appeared numerically more frequent in limb lesions, this association did not reach statistical significance (Fisher’s exact test, *p* = 0.182).

**Figure 2:**
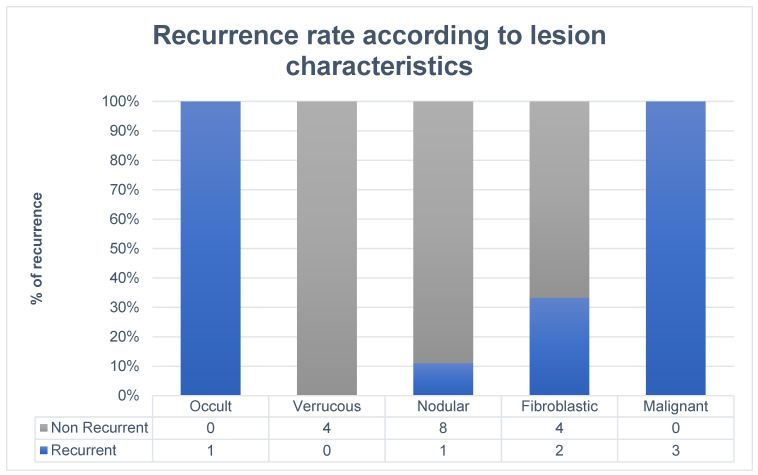
Distribution of recurrent and non-recurrent lesions according to clinical sarcoid subtype. A total of 23 recurrent equine sarcoid lesions were classified as occult (n = 1), verrucous (n = 4), nodular (n = 9), fibroblastic (n = 6), or malignant (n = 3). The figure shows the number of lesions within each clinical subtype that remained free from recurrence and those that developed recurrence during the follow-up period after treatment with surgical cytoreduction and intraoperative ECT.

Clinical appearance appeared to show a possible association to influence treatment outcome. All malignant sarcoids (3/3) recurred during follow-up, whereas recurrence was observed in 2 of 6 fibroblastic lesions, 1 of 9 nodular lesions, and the single occult lesion included in the study. No recurrence was recorded among verrucous sarcoids. When malignant lesions were compared with all other clinical subtypes combined, a significant association with recurrence was identified (Fisher’s exact test, *p* = 0.020). However, this finding should be interpreted cautiously due to the limited number of malignant lesions included in the study. When fibroblastic and malignant lesions were grouped together as clinically aggressive forms, recurrence was observed in 5 of 9 lesions (55.6%), compared with 2 of 14 lesions (14.3%) classified as nodular, verrucous, or occult. This association approached statistical significance but did not reach the predefined threshold (Fisher’s exact test, *p* = 0.066) (Figure 2).

### 3.5. Recurrence According to Treatment Variables

Recurrence occurred in 4 of 11 lesions (36.4%) treated with carboplatin and in 3 of 12 lesions (25.0%) treated with cisplatin. No significant association was identified between the chemotherapeutic agent used and recurrence rate (Fisher’s exact test, *p* = 0.667). Similarly, recurrence was observed in 3 of 15 lesions (20.0%) without documented previous treatment history and in 4 of 8 lesions (50.0%) with one or more recorded previous treatment attempts. Although recurrent disease appeared numerically more common among previously treated lesions, this difference did not reach statistical significance (Fisher’s exact test, *p* = 0.182). Given the limited number of lesions included in each treatment group, this comparison should be regarded as exploratory and does not allow conclusions regarding the comparative efficacy of the two platinum compounds.

### 3.6. Association Between Lesion Size and Recurrence

Lesion size tended to be greater among RES than among lesions that remained locally controlled. The median maximum diameter of recurrent lesions was 9.5 cm (range 1.5–25.0 cm), compared with 4.5 cm (range 2.0–11.0 cm) in non-recurrent lesions. Despite this apparent difference, statistical analysis did not identify a significant association between lesion size and recurrence (Mann–Whitney U test, *p* = 0.158).

Associations between selected lesion- and treatment-related variables and local recurrence are presented in Table 2.

## 4. Discussion

The present study evaluated the clinical outcome of surgical cytoreduction combined with intraoperative ECT in a population composed exclusively of RES. This aspect represents one of the principal strengths of the study and distinguishes it from many previously published reports investigating ECT in equine oncology. Whereas most available studies include heterogeneous populations consisting of both primary and recurrent lesions [12,15,18], all sarcoids included in the present investigation had previously undergone at least one therapeutic intervention and subsequently recurred.

Overall, local tumour control was achieved in the majority of treated lesions. Recurrence was observed in 7 of 23 lesions, corresponding to a lesion-based recurrence rate of 30.4%, while 69.6% of lesions remained free from recurrence throughout the available follow-up period. At the horse level, recurrence of at least one lesion was documented in 35.7% of horses. Although these figures should be interpreted cautiously because of the limited sample size and retrospective design, they appear clinically encouraging when considered in the context of recurrent disease.

Comparison among studies investigating ES treatments is notoriously difficult because of substantial variation in lesion selection, clinical subtype distribution, anatomical location, follow-up duration, and outcome definitions. Nevertheless, recurrence rates reported following conventional surgical excision alone remain highly variable and may exceed 50% in certain populations, particularly when lesions are incompletely excised or located in anatomically challenging regions [15,21]. Similar variability has been reported for cryotherapy, laser excision, topical cytotoxic therapies, and immunotherapeutic approaches [15]. As emphasized by Hollis (2023) [3], recurrence remains one of the defining clinical characteristics of sarcoid disease regardless of treatment modality, particularly in lesions that have already demonstrated biological persistence following previous interventions.

Additionally, the approximately 30% local recurrence rate observed in the present study should be interpreted in light of several factors that could not be controlled after hospital discharge. Once horses returned to their usual environment, several factors—including owner compliance, wound management, environmental conditions, insect exposure, and individual host factors—may have influenced healing and long-term local tumour control. Consequently, recurrence likely reflects the interaction of treatment-related, biological, and environmental factors rather than the efficacy of the ECT protocol alone. In addition, biting flies have been proposed as mechanical vectors of BPV, potentially contributing to viral persistence or local reinoculation after treatment [22,23].

Surgical cytoreduction reduces tumour burden by removing grossly visible disease, whereas ECT targets residual neoplastic cells that may remain beyond clinically identifiable margins [24,25]. This approach is particularly relevant in recurrent sarcoids, where fibrosis, chronic inflammation and tissue distortion following previous treatments often preclude complete excision [26]. These changes may also reduce homogeneous distribution of the chemotherapeutic agent within the surgical bed, potentially limiting treatment efficacy. Although this hypothesis could not be investigated in the present study, it represents a plausible explanation for the greater therapeutic challenge posed by recurrent lesions [27].

ECT enhances intracellular uptake of chemotherapeutic agents through reversible electroporation while inducing a transient vascular-lock effect, thereby increasing local cytotoxicity with minimal systemic exposure [18,20]. Current veterinary guidelines support ECT as a safe and effective local treatment for superficial tumours and support its use either as a primary treatment or as an adjunctive modality following cytoreduction [16,17].

One of the most interesting observations of the present study was the trend towards different recurrence rates among clinical subtypes. Although these findings should be interpreted cautiously given the limited sample size and the exploratory nature of the statistical analyses, all malignant sarcoids included in the study recurred during follow-up, whereas recurrence was less frequent among nodular and verrucous lesions. According to the “Equine Sarcoid Consensus Statement” (2026), malignant lesions represent the most aggressive end of the sarcoid spectrum and are characterised by extensive local invasion, multifocal extension, and a marked tendency toward progression despite treatment [2,28]. Similarly, fibroblastic lesions showed higher recurrence rates than nodular and verrucous forms, consistent with previous reports suggesting a more locally aggressive biological behaviour, particularly following repeated trauma or unsuccessful treatment attempts [28,29]. The tendency observed in the present study for fibroblastic and malignant lesions to recur more frequently than other clinical types therefore appears consistent with current sarcoids bibliography.

Anatomical location also appeared to influence treatment outcome. Limb lesions showed a numerically higher recurrence rate than lesions located elsewhere, although statistical significance was not achieved [30]. Sarcoids located on the limbs are generally considered more difficult to treat because restricted surgical access, limited tissue availability for closure, continuous motion, and repeated mechanical irritation may adversely affect wound healing and local tumour control [31]. While the limited sample size precludes definitive conclusions, this observation is consistent with previous studies identifying anatomical location as an important prognostic factor in sarcoid management [32].

Lesion size also appeared to show a trend towards an association with recurrence. Recurrent lesions were, on average, larger than lesions that remained locally controlled, with median diameters of 9.5 cm and 4.5 cm, respectively. Although this difference did not reach statistical significance, the observed trend is clinically relevant. Larger lesions may contain greater tumour burden, more extensive microscopic infiltration, and a broader field of transformed fibroblasts extending beyond visible margins. In addition, larger lesions often require more extensive surgical cytoreduction and may be associated with larger postoperative defects, potentially increasing the complexity of local tumour control [33]. The absence of statistical significance in the present study may simply reflect the limited number of lesions available for analysis rather than a true lack of biological association.

No significant difference in recurrence rate was identified between lesions treated with cisplatin and those treated with carboplatin. Interpretation of this finding requires caution because the study was not designed or powered to compare chemotherapeutic agents directly. Nevertheless, both platinum compounds are commonly used in veterinary oncology and share similar mechanisms of action involving DNA cross-linking and inhibition of cellular replication [34,35]. The comparable outcomes observed in the present study should not be interpreted as evidence of equivalent efficacy between cisplatin and carboplatin. Rather, they indicate that no obvious differences were observed within this limited cohort. Larger prospective studies specifically designed to compare the two drugs are required before any conclusions regarding comparative efficacy can be drawn.

In the present study, all horses underwent a single planned intraoperative ECT session irrespective of lesion characteristics. This approach was adopted to standardize treatment and because evidence-based recommendations regarding the optimal number of ECT sessions in RES are currently lacking. However, the distribution of recurrences observed among the different clinical subtypes suggests that a single treatment session may not be equally adequate for all lesions. In particular, nodular sarcoids appeared to achieve durable local control more consistently, whereas occult and malignant lesions showed a greater tendency to recur. Although the limited number of cases precludes definitive conclusions, these findings raise the possibility that treatment protocols could eventually be tailored according to sarcoid subtype, with more aggressive lesions potentially benefiting from planned additional ECT sessions. Further prospective studies are required to determine whether repeated treatments improve long-term local control in selected lesion types.

Although direct comparison among studies should be interpreted cautiously, the outcomes observed in the present study compare favourably with those reported for other treatment modalities used for ES. Cryosurgery has historically been considered an effective option for selected lesions, particularly when combined with surgical debulking; however, recurrence rates remain variable and are influenced by lesion location, clinical subtype, and completeness of treatment [36,37,38]. Similarly, immunotherapeutic approaches, including Bacillus Calmette–Guérin (BCG) immunotherapy [39,40], Baypamun inactivated parapoxovis virus [41], and various other immunotherapic approaches [42] have demonstrated encouraging results, but are associated with inconsistent responses and occasional severe inflammatory reactions. Topical [43] and intralesional chemotherapeutic protocols [44] may also achieve satisfactory local control in some cases, although recurrence remains a common challenge, particularly in previously treated lesions.

The combination of surgical cytoreduction and ECT may offer several practical advantages in this setting. Unlike cryotherapy, which relies on adequate freezing of the entire treatment field, or immunotherapy, which depends on host immune responsiveness, ECT provides a direct local cytotoxic effect while minimizing systemic drug exposure. Furthermore, the ability to treat irregular surgical beds and areas where wide surgical margins cannot be achieved may be particularly advantageous in recurrent lesions and anatomically challenging locations.

An additional clinically relevant observation concerns postoperative wound management. All lesions were managed by second-intention healing following treatment, and no major postoperative complications requiring surgical revision were identified. The decision not to perform primary closure was intentional and reflected both the size of many surgical defects and the concern that local administration of chemotherapeutic agents combined with electroporation could adversely affect early wound healing [45,46].

Although no treatment-related complications directly attributable to ECT were observed in the present study, the procedure requires general anaesthesia, which represents an inherent source of morbidity and mortality in horses independent of the oncological treatment itself. This aspect should be considered when evaluating the overall risk–benefit profile of the procedure, particularly in horses requiring repeated interventions [47].

The present study has several limitations that should be acknowledged. First, its retrospective design inherently limited control over data collection and follow-up procedures. Second, the number of horses and lesions included was relatively small, reducing statistical power and increasing the risk of type II error [48]. An additional methodological consideration concerns the lesion-based statistical analyses. Although multiple lesions originating from the same horse cannot be considered fully independent observations, lesion-level analyses were intentionally performed because treatment decisions and prognostic assessment in ES are largely based on lesion-specific characteristics, such as anatomical location, clinical subtype and lesion size. Analysing the horse as the sole unit of observation would not have allowed exploration of these lesion-specific variables. To provide a complementary perspective, treatment outcomes were also evaluated at the horse level. Nevertheless, the potential effect of intra-animal correlation should be considered when interpreting the exploratory statistical analyses, and the observed associations should be regarded as hypothesis-generating rather than confirmatory. Third, although all lesions were known to have undergone at least one previous therapeutic intervention, detailed information regarding the exact nature and number of previous treatments was not consistently available. Consequently, this incomplete documentation may also represent a source of bias, as differences in the type, number and timing of previous treatments could have influenced local tissue architecture, biological behaviour of the lesions, and ultimately treatment response.

Additional limitations include heterogeneity in lesion location, clinical subtype, and follow-up duration, all of which may have influenced the observed results.

Despite these limitations, the study provides clinically relevant information regarding the management of RES, a population that remains underrepresented in the literature despite representing one of the most challenging groups encountered in daily practice. The results suggest that surgical cytoreduction combined with intraoperative ECT can provide satisfactory local tumour control in many recurrent lesions and may represent a valuable therapeutic option when previous treatments have failed.

Although the present study demonstrated a lower local recurrence rate than that reported in many previous studies, approximately 30% of lesions still developed local recurrence. These findings suggest that there remains considerable scope for improving current treatment protocols. In particular, the distribution of recurrences observed among the different clinical sarcoid subtypes raises the possibility that a uniform treatment approach may not be optimal for all lesions. Although the limited sample size precludes definitive conclusions, lesion subtype appeared to have a greater influence on clinical outcome than other evaluated variables, suggesting that future treatment protocols may benefit from a more individualized approach.

At present, no standardized evidence-based protocol exists to guide the optimal ECT regimen for different clinical forms of RES. Further prospective studies are therefore warranted to determine whether treatment protocols should be tailored according to lesion characteristics, including clinical subtype, by optimizing variables such as the chemotherapeutic dose, timing of treatment, wound management, and the need for planned repeated ECT sessions. Such an individualized approach may further improve local tumour control and reduce recurrence rates beyond those achieved with a single standardized treatment protocol.

Rather than seeking a single optimal treatment protocol for all RES, future research should aim to identify lesion-specific therapeutic strategies capable of maximizing local tumour control while minimizing unnecessary interventions.

## 5. Conclusions

Surgical cytoreduction combined with intraoperative ECT provided satisfactory local tumour control in the majority of RES included in this study. Considering that all lesions had previously failed at least one therapeutic intervention, the recurrence rate observed may be regarded as clinically encouraging and supports the use of this combined approach in challenging cases of treatment-resistant disease.

Clinical subtype and lesion size appeared to influence treatment outcome, with malignant and fibroblastic lesions showing a greater tendency toward recurrence and larger lesions demonstrating less favourable outcomes. Although these associations require confirmation in larger populations, they may represent clinically relevant factors when selecting treatment strategies and counselling owners regarding prognosis.

The results of the present study suggest that ECT can be successfully integrated into the management of RES following surgical cytoreduction and may represent a valuable addition to the therapeutic options currently available for equine oncology. Further prospective multicentre studies with larger case numbers, standardized protocols, and longer follow-up periods are warranted to better define prognostic factors and optimize treatment recommendations.

## Figures and Tables

**Table 1:** Signalment, lesion characteristics, and treatment outcome of horses included in the study. Fourteen horses presenting a total of 23 recurrent equine sarcoid lesions were included. Information reported includes breed, sex, age at treatment, number of lesions per horse, anatomical location, clinical subtype, maximum lesion diameter, chemotherapeutic agent administered during ECT, and clinical outcome. All lesions had previously undergone at least one therapeutic intervention before referral and were therefore classified as recurrent. NR = no recurrence; R = recurrence.

Case	Breed	Sex	Age (Years)	Number of Lesions	Anatomical Location(s)	Clinical Subtype(s)	Maximum Lesion Diameter (cm)	Chemotherapeutic Agent(s)	Outcome
1	Purebred Arabian	Mare	14	1	Ear	Fibroblastic	5.0	Carboplatin	NR
2	Mixed-breed horse	Gelding	6	1	Head	Nodular	3.0	Cisplatin	NR
3	Quarter Horse	Gelding	15	1	Genitomammary	Verrucous	3.0	Carboplatin	NR
4	Paint Horse	Gelding	8	1	Genitomammary	Verrucous	3.0	Cisplatin	NR
5	Italian Warmblood	Gelding	12	Multiple	Multiple	Nodular	3.0	Carboplatin	R
6	Selle Français	Gelding	9	Multiple	Multiple	Verrucous	6.5	Carboplatin	NR
7	Purebred Arabian	Mare	14	Multiple	Pelvic limb	Nodular	6.0	Carboplatin	NR
8	Appaloosa	Mare	8	Multiple	Pelvic limb	Fibroblastic/Malignant	10.0	Carboplatin	R
9	Mixed-breed horse	Mare	6	Multiple	Periocular	Nodular	3.0	Carboplatin	NR
10	Haflinger	Mare	17	Multiple	Pelvic limb	Fibroblastic	11.0	Cisplatin	NR
11	Haflinger	Mare	21	Multiple	Pelvic limb	Fibroblastic	9.5	Cisplatin	R
12	Italian Warmblood	Mare	7	Multiple	Thoracic limb	Nodular	5.0	Cisplatin	NR
13	Italian Warmblood	Gelding	5	Multiple	Neck	Not available	3.0	Cisplatin	NR
14	KWPN	Mare	20	Multiple	Genitomammary	Fibroblastic	12.0	Cisplatin	R

**Table 2:** Association between selected lesion and treatment variables and local recurrence. Recurrence rates are presented for each category together with the corresponding *p*-values. Associations between categorical variables and recurrence were evaluated using Fisher’s exact test, whereas lesion size was compared between recurrent and non-recurrent lesions using the Mann–Whitney U test. Given the retrospective study design and limited sample size, all statistical analyses should be considered exploratory.

Variable	Category	Recurrence n/N (%)	*p* Value
Anatomical location	Limb	4/8 (50.0)	0.182
	Non-limb	3/15 (20.0)	
Clinical subtype	Malignant	3/3 (100)	0.020
	Other	4/20 (20.0)	
	Fibroblastic + malignant	5/9 (55.6)	0.066
	Other	2/14 (14.3)	
Chemotherapeutic agent	Cisplatin	3/12 (25.0)	0.667
	Carboplatin	4/11 (36.4)	
Lesion size	Median (range)	9.5 vs. 4.5 cm	0.158

## Data Availability

The data presented in this study are available on request from the corresponding author. The data are not publicly available due to privacy restrictions.

## Abbreviations

The following abbreviations are used in this manuscript:

BPV	Bovine Papillomavirus
ECT	Electrochemotherapy
ES	Equine Sarcoid(s)
RES	Recurrent Equine Sarcoid(s)
IV	Intravenous
PO	Per os (oral administration)
TNCC	Total Nucleated Cell Count
SD	Standard Deviation
CI	Confidence Interval
